# Inverted optical intrinsic response accompanied by decreased cerebral blood flow are related to both neuronal inhibition and excitation

**DOI:** 10.1038/srep21627

**Published:** 2016-02-10

**Authors:** Zengguang Ma, Pengjia Cao, Pengcheng Sun, Linna Zhao, Liming Li, Shanbao Tong, Yiliang Lu, Yan Yan, Yao Chen, Xinyu Chai

**Affiliations:** 1 School of Biomedical Engineering, Shanghai Jiao Tong University, Shanghai 200240, China; 2 Institute of Neuroscience and State Key Laboratory of Neuroscience, Shanghai Institutes for Biological Sciences, Chinese Academy of Sciences, Shanghai 200031, China

## Abstract

Negative hemodynamic response has been widely reported in blood oxygenation level-dependent (BOLD) functional magnetic resonance imaging studies, however its origin is still controversial. Optical intrinsic signal (OIS) imaging can be used to study brain activity by simultaneously recording hemodynamic signals at different wavelengths with high spatial resolution. In this study, we found transcorneal electrical stimulation (TcES) could elicit both positive OIS response (POR) and negative OIS response (NOR) in cats’ visual cortex. We then investigated the property of this negative response to TcES and its relationship with cerebral blood flow (CBF) and neuronal activity. Results from laser speckle contrast imaging showed decreased CBF in the NOR region while increased CBF in the POR region. Both planar and laminar electrophysiological recordings in the middle (500–700 μm) cortical layers demonstrated that decreased and increased neuronal activities were coexisted in the NOR region. Furthermore, decreased neuronal activity was also detected in the deep cortical layers in the NOR region. This work provides evidence that the negative OIS together with the decreased CBF should be explained by mechanisms of both neuronal inhibition and excitation within middle cortical layers. Our results would be important for interpreting neurophysiological mechanisms underlying the negative BOLD signals.

There is a tight coupling between changes in cerebral neuronal activity and resultant hemodynamic responses^1^,^2^,^3^. Generally, an increase in neuronal activity promotes oxygen consumption, and thus demands more cerebral blood flow (CBF) in that brain region. Most neuroimaging techniques, including functional magnetic resonance imaging (fMRI) and optical intrinsic signal (OIS) imaging, use these hemodynamic changes as surrogates for neuronal activities in the brain. Besides commonly reported positive hemodynamic response, which has been shown to correlate with enhancement of the neuronal activity^1^,^4^, a negative hemodynamic response has received much attention recently^5^,^6^,^7^,^8^,^9^,^10^,^11^. Understanding the relationship that links neuronal activities and hemodynamic signals is particularly important for accurate interpretation of the results from these imaging studies.

The negative hemodynamic response has been found in somatosensory and motor cortices by physical stimulation. By using blood oxygenation level-dependent (BOLD) fMRI, some groups showed that the negative BOLD response in humans’ ipsilateral motor cortex while they performing a right-hand pinch grip represented a marker of neuronal deactivation^12^,^13^. By using OIS imaging, in 2005 Devor *et al.*^14^ found that the negative hemodynamic response in rodent somatosensory cortex responding to single-whisker deflection did not correspond to observable changes in neuronal activity. Furthermore, in 2008 Devor *et al.*^15^ demonstrated that the negative hemodynamic response to electrical stimulation of the whisker pad was accompanied by an increase in neuronal spiking and glucose consumption. On the contrary, other groups concluded that the negative hemodynamic response to electrical stimulation reflected a functionally effective inhibition^16^,^17^,^18^ or neuronal inhibition^7^,^19^,^20^ in the somatosensory system.

In visual system, the negative hemodynamic response has also been widely investigated by using visual stimulation. Harel *et al.*^21^ concluded that the negative BOLD response to a moving grating pattern was caused by a redistribution of CBF from the less active regions to the most CBF-demanding areas. A growing body of research demonstrated that the negative BOLD response was associated with neuronal inhibition^22^,^23^,^24^,^25^,^26^. Besides, the negative BOLD response could be accounted for by heightened neuronal activity and oxygen consumption without sufficient CBF increment^27^. Moreover, some studies showed that although the negative BOLD response was neuronal in origin, neurovascular coupling mechanisms underlying the negative and positive BOLD responses were not only different from each other but also cortical layer dependent^10^,^28^,^29^. Despite plenty of studies on the negative hemodynamic response to visual stimulation have been performed, rare studies have been focused on this response to electrical stimulation of visual pathway. By electrically stimulating the lateral geniculate nucleus (LGN) in monkeys, Logothetis *et al.*^30^ found a positive BOLD response in the regions of primary visual cortex receiving direct input from the stimulated LGN site, while a negative BOLD response in the retinotopically matched regions of extrastriate cortex. They concluded that cortico-cortical signal propagation was disrupted by electrical stimulation, and the reduction in BOLD response was likely a result of synaptic inhibition. This was further confirmed by the negative BOLD response detected in anterior intraparietal area through electrical stimulation of V5/MT and surrounding areas^31^.

In summary, the negative hemodynamic response could be explained by several independent mechanisms, such as an increase in neuronal activity and oxygen consumption without sufficient CBF increases, a decrease in CBF due to neural inhibition, a reduction in CBF due to vasoconstriction without neural inhibition, a decrease of CBF in the superficial cortical layers accompanied by an increase of cerebral blood volume (CBV) in the deeper layers, a pure hemodynamic effect, etc.^2^,^5^,^6^,^32^. So far, its mechanisms are still controversial and need further study.

The negative BOLD response was investigated by using concurrent fMRI and OIS imaging^33^. Due to its high spatial resolution, multi-wavelength OIS imaging could play an important role in investigating mechanisms underlying the negative hemodynamic response by simultaneously mapping hemodynamic signals at different wavelengths^34^. Moreover, electrophysiological recording which reflects the neural activity directly should be performed to explore changes of neuronal activity when the negative hemodynamic response is found. Planar electrophysiological recordings performed by Utah Electrode Array (UEA) can offer multi-channel recordings simultaneously^35^, which may provide information of the origin of the negative hemodynamic response. While linear microelectrode array (LMA) can record neural activities in different cortical layers, which could also provide some indications about the mechanisms underlying the negative hemodynamic response. Both of them could help us to investigate the differences of neuronal activity between cortical areas with the positive and negative hemodynamic responses.

Our previous work found transcorneal electrical stimulation (TcES) through an ERG-jet corneal electrode could consistently elicit hemodynamic responses in cats’ visual cortex, using multi-wavelength OIS recording^36^. In the present study we found an inverted OIS response to TcES in cats’ visual cortex. To the best of our knowledge, this is the first time that antagonistic cortical activation evoked by electrical stimulation of intact retina is reported. We then further investigated the spatiotemporal patterns of this cerebral antagonistic response and its relationship to TcES. In order to explore potential mechanisms of the inverted OIS response, we evaluated CBF changes using laser speckle contrast imaging (LSCI) and recorded local neuronal activity using planar and laminar electrophysiological recordings. Studying the origin of antagonistic cortical activation induced by retinal electrical stimulation not only provides useful insights into retinocortical signal transduction and neurophysiological underpinnings underlying prosthetic vision, but also promotes understanding of mechanisms accounting for the widely discussed negative BOLD response. Our results suggest that both inhibitory and excitatory mechanisms should be incorporated to interpret the inverted OIS response.

## Results

### Spatial patterns of antagonistic activation by TcES

**Figure 1:**
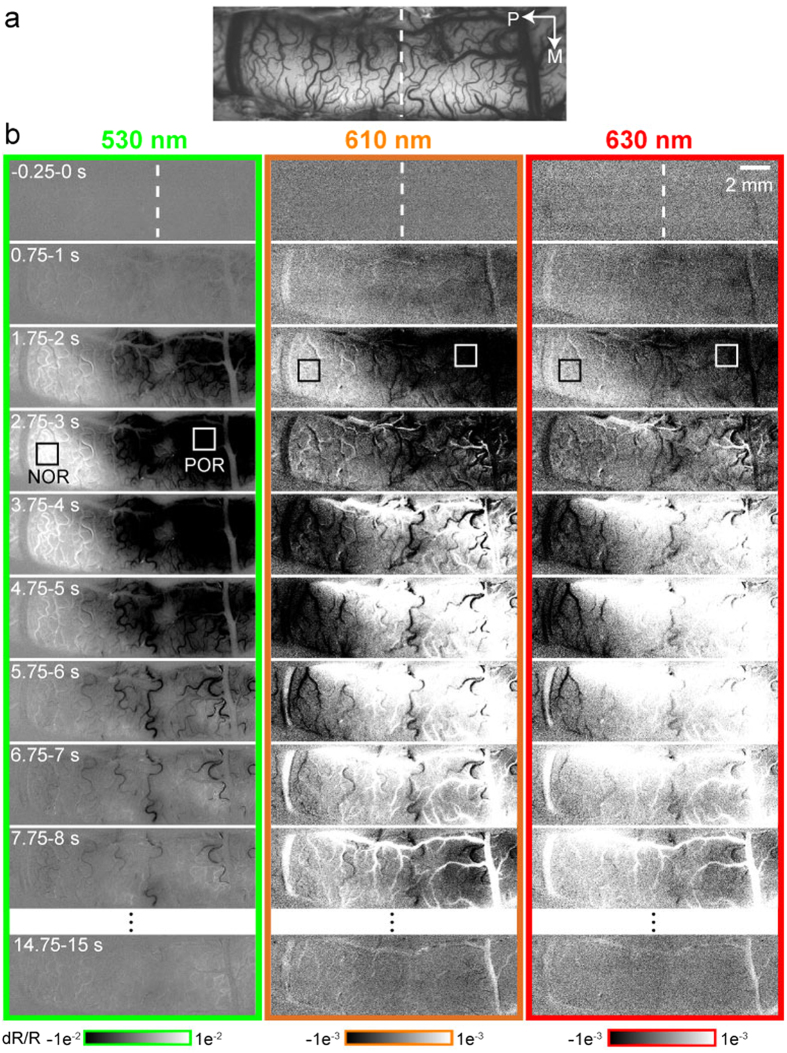
Spatiotemporal properties of cortical activation by TcES (20 Hz, 1.2 mA, 10 ms) in one experiment. (**a**) Blood vessel map obtained under 530-nm illumination. (**b**) The three columns display high-resolution imaging of evoked cortical intrinsic signals under 530- (green panel), 610- (orange panel) and 630-nm (red panel) illumination, respectively. Data were averaged across 160 trials. Stimulus started at 0 s and lasted for 2 s. White and black boxes indicate two ROIs located at the POR and NOR regions for subsequent analysis. Color bars at the bottom represent reflectance change (dR/R). Vertical lines indicate the Horsley–Clarke coordinates AP0 (also shown in Figs 3, 4, 5, 6). P, posterior; M, medial.

Figure 1a shows an image of the blood vessels in the imaged area. Figure 1b displays the evolution of optical responses to the TcES. Hemodynamic signals at volumetric (530 nm) and oximetric (610 and 630 nm) wavelengths in the contralateral visual cortex after the TcES exhibited a clear antagonistic spatial pattern of activation in 26 out of 31 cats. The typical OIS consisted of a volumetric component, which monophasically decreased, and an oximetric component, which had an initial dip followed by an overshoot. Hereafter, this pattern is referred to as the ‘positive OIS response’ (POR). In adjacent cortical areas, an inverted OIS response was observed. It included an increase in the volumetric component and an initial increase and subsequent decrease in the oximetric component. We refer to this as the ‘negative OIS response’ (NOR). The NOR region was posterior to the POR region. The monophasic changes in volumetric signals of both NOR and POR regions peaked at about 2.75–3 s. However, the oximetric signals of the POR had an initial dip at 1.75–2 s followed by a strong overshoot at 4.75–5 s, whereas the oximetric signals of the NOR comprised an initial increase at 1.75–2 s and a subsequent big decrease in light reflection at 4.75–5 s. Compared to the volumetric signal, the strength of the oximetric signal was much weaker.

### Temporal patterns of antagonistic activation by TcES

**Figure 2:**
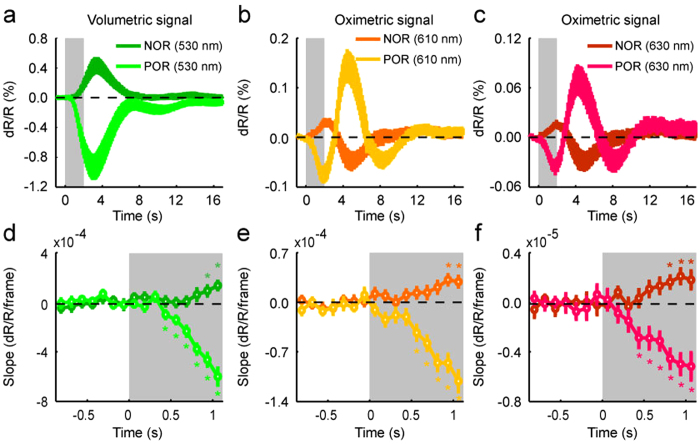
Temporal patterns of antagonistic activation by TcES (20 Hz, 1.2 mA, 10 ms). (**a**−**c**) Temporal profiles of volumetric and oximetric signal changes after TcES averaged across 16 cats. (**d**−**f**) The slopes of reflectance changes for volumetric and oximetric responses. *indicates slopes significantly different from response baseline (Wilcoxon test, *P* < 0.05, n = 16). The gray rectangles denote the stimulus duration, which also shown in Figs 3, 4, 5. Error bars show SEM.

To quantitatively compare properties of the volumetric and oximetric signals between the POR and NOR regions, time courses of reflectance change responding to TcES (20 Hz, 1.2 mA, 10 ms) were extracted from two ROIs (e.g. white and black boxes in Fig. 1b), which were separately located in the POR and NOR regions (see Methods). The volumetric and oximetric signals of POR elicited by TcES have been previously described^36^. Monophasic volumetric signal of NOR had an increase peaked at 3.4 ± 0.3 s, which was significantly later than the POR peak time at 3.1 ± 0.1 s (Wilcoxon test, *P* = 0.017, n = 16), with only half of the reflectance change of that in the POR region (0.429 ± 0.093% vs. −0.932 ± 0.151%, Fig. 2a). Temporal profile of oximetric signal was triphasic in the POR region and biphasic in the NOR region (Fig. 2b,c). Initially, oximetric signal at 610 nm of the POR decreases rapidly to a minimum of −0.080 ± 0.013% at 1.9 s while that of the NOR increased to 0.030 ± 0.006% with a latency of 2.2 s. After this transient response, oximetric signal of the POR rose to a second peak of 0.147 ± 0.027% with a latency of 4.4 s while that of the NOR decreased to −0.049 ± 0.014% at 4.8 s. The time courses of 630-nm signals were similar to those of 610-nm signals. The POR signal declined fast to a minimum of −0.039 ± 0.006% with a latency of 1.8 s while the NOR signal rose briefly to a maximum of 0.017 ± 0.003% at 1.9 s. The second peak amplitude of the POR was 0.071 ± 0.016% at 4.4 s while that of the NOR was −0.030 ± 0.009% at 4.9 s. The first peak of the oximetric signal in the POR region appeared significantly earlier than that in the NOR region (Wilcoxon test, *P* = 0.007 at 610 nm, *P* = 0.013 at 610 nm, n = 16). Quantifying onset latency as the first time point where the response displayed a statistically significant (Wilcoxon test, *P* < 0.05, n = 16) inflection in slope demonstrated that response onsets of the POR at all wavelengths were shorter than those of the NOR (POR: 0.4 s at 530 nm, 0.6 s at 610 nm, 0.4 s at 630 nm; NOR: 0.9 s at 530 nm, 0.9 s at 610 nm, 0.8 s at 630 nm) (Fig. 2d–f). Our results also showed that the onset latency of the volumetric signal was as fast as that of the oximetric signal, either in the POR or the NOR region.

Our results confirmed that both the oximetric signals at 610 and 630 nm showed similar spatiotemporal patterns of the antagonistic response, however, the signal strength at 630 nm was weaker than that at 610 nm. Therefore, the 630-nm signal was excluded from following analysis.

### Effect of electrical stimulation on NOR

**Figure 3:**
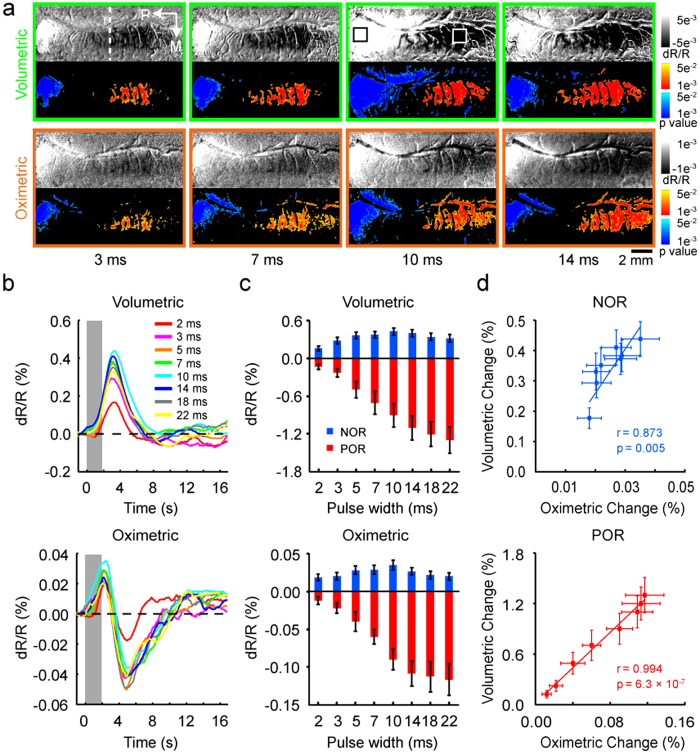
Dependence of cortical OIS responses on stimulus pulse width. (**a**) Optical single-condition maps averaged over 1.75–2.25 s after stimulus onset and corresponding *P*-value maps (two-tailed t test, *P* < 0.05, n = 148 trials) of cortical activation evoked by various stimulus pulse widths. Top: 530-nm images; Bottom: 610-nm images. Color bars for single-condition maps represent reflectance change (dR/R). Color bars for *P*-value maps indicate *P*-values for decreases (warm colors, POR) and increases (cold colors, NOR) in light reflectance. (**b**) Averaged time courses of volumetric and oximetric signal changes evoked by stimuli of different pulse widths in the NOR region. (**c**) Peak values of the POR and NOR signal changes as a function of stimulus pulse widths. Data were averaged over all of the pixels within selected ROIs across 6 cats. (**d**) Oximetric and volumetric signals (the absolute value of magnitude) were significantly related to each other in both POR and NOR regions. Error bars indicate SEM.

Since the amplitudes and activated areas of the positive OIS responses were correlated with the electrical stimulation^36^, a question arises whether the negative responses are also related to the electrical stimulation. We investigated this question in 6 cats by analyzing the optical signals at wavelengths of 530 and 610 nm with pulse widths of TcES varying from 2 to 22 ms at 20 Hz and 1.2 mA. Spatial extent of activation areas as *P*-value maps for the POR (warm colors) and the NOR (cold colors) in one experiment were displayed paired with their relevant grayscale maps (Fig. 3a; see Methods). The images averaged between 1.75–2.25 s under four different stimulus pulse widths were displayed to demonstrate the response changes. In agreement with our previous studies, increasing the pulse width of TcES led to an increase of the positive activation area. On the contrary, the negative activation area enlarged at first and then diminished with the increase of pulse width. Figure 3b shows time courses of the volumetric and oximetric responses at different stimulus pulse widths in the NOR region. The averaged response magnitudes at peaks as a function of pulse widths were plotted in Fig. 3c. With the increment of pulse width, both of the oximetric and volumetric responses in the POR region were monotonically increased while those in the NOR region were unimodal, peaking at pulse width of 10 ms. The relationships between the amplitudes of oximetric and volumetric signals were significantly related in both the POR and NOR regions (Fig. 3d, POR, r = 0.994, *P* = 6.3 × 10^−7^; NOR, r = 0.873, *P* = 0.005).

### Local cerebral blood flow measured by laser speckle contrast imaging

**Figure 4:**
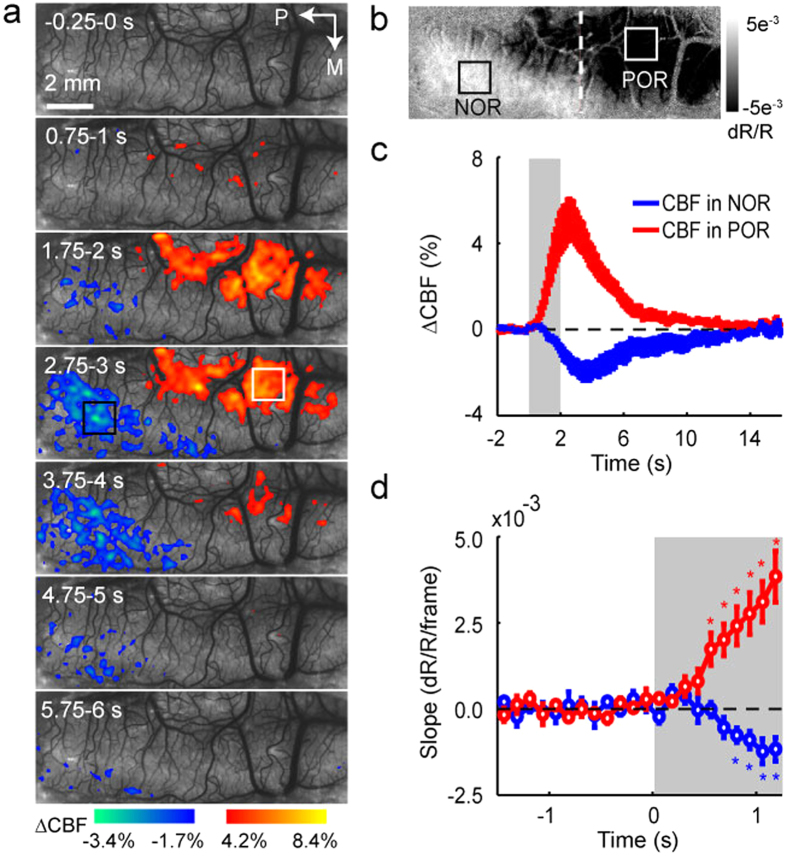
Relative changes in CBF responding to TcES (20 Hz, 1.2 mA, 10 ms). (**a**) High spatiotemporal resolution imaging of CBF averaged over 96 trials from one cat. Stimulus started at 0 s and lasted for 2 s. White and black boxes indicate two ROIs determined by OIS imaging (shown in (b)) located at the POR and NOR regions for subsequent analysis. Color bars represent relative changes in CBF. (**b**) Map of volumetric signal averaged from 1.75–2.25 s by OIS imaging from the same cat. (**c**) The graph illustrates the percent changes in CBF in the POR and NOR regions. (**d**) The slopes of reflectance changes for CBF. *indicates slopes significantly different from baseline before stimulus onset (Wilcoxon test, *P* < 0.05, n = 5). The data in (**c**,**d**) were averaged over 5 cats. Error bars show SEM.

Functional imaging of visual cortex with LSCI was performed to provide a two-dimensional real-time, full-field imaging of CBF with high spatiotemporal resolution. We recorded the hemodynamic signals responding to TcES with the OIS imaging then directly measured the CBF changes with the LSCI in 5 cats. A time series of maps from one cat are shown at 1-s intervals in Fig. 4a, which revealed that areas of increased and decreased CBF coexisted in the visual cortices following TcES. Pixels whose value representing relative CBF change greater than half of its positive peak (warm colors) and less than half of its negative peak (cold colors) are color-coded and superimposed on the speckle contrast images. CBF in the POR region revealed by the OIS imaging (Fig. 4b) was increased, while CBF in the NOR region was decreased. Corresponding time courses of the CBF changes were extracted from all pixels within two ROIs determined by the OIS imaging (Fig. 4b, black and white boxes) and averaged across 5 cats. TcES elicited a large CBF increase in the POR region and a small CBF decrease in the NOR region (Fig. 4c). The time-to-peak of decreased CBF in the NOR region was at 3.9 ± 0.3 s after the stimulus onset, which was significantly (Wilcoxon test, *P* = 0.031, n = 5) longer than the peak time at 2.7 ± 0.1 s of increased CBF in the POR region. Quantifying onset latency of the CBF change as the first time point where the CBF displayed a statistically significant (Wilcoxon test, *P* < 0.05, n = 5) inflection in slope demonstrated that the onset latency of the CBF change in the NOR region was also later than that in the POR region (NOR, 0.8 s; POR, 0.6 s) (Fig. 4d).

### Planar LFP and MUA recordings by Utah microelectrode array

**Figure 5:**
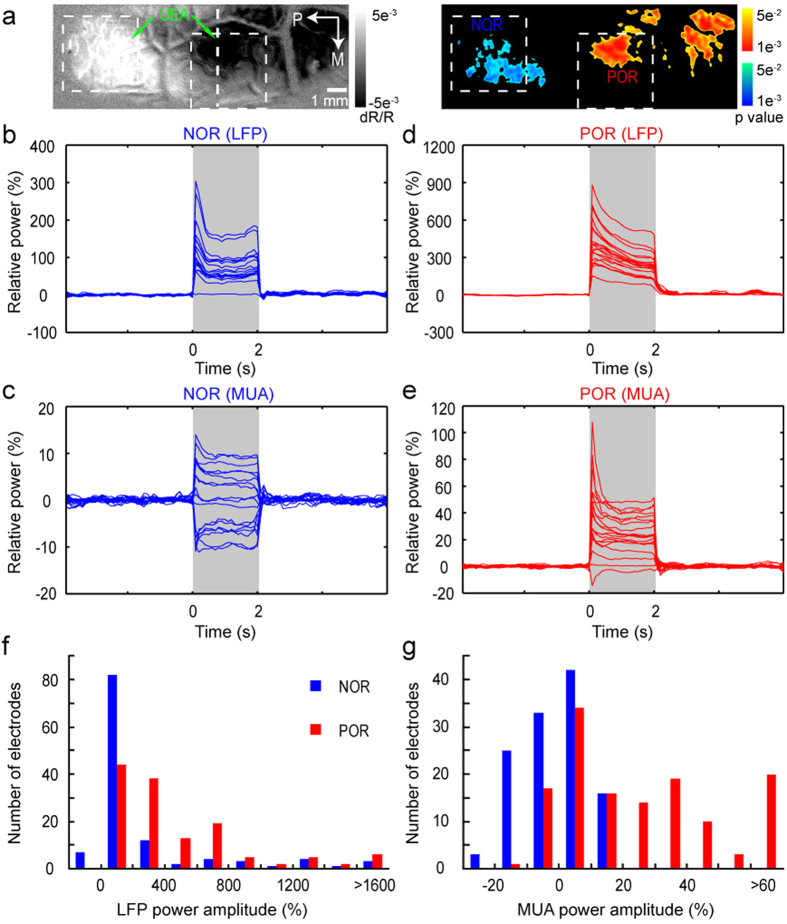
UEA recordings of MUA and LFP by TcES (10 Hz, 1.2 mA, 3 ms) in one cat. (**a**) Left: optical imaging map (530 nm) and the schematic illustration of the UEA penetrated into the cortical surface; Right: *P*-value map (two-tailed t test, *P* < 0.05, n = 148 trials) of cortical activation. (**b**−**e**) 30–130 Hz (LFP) and 300–2500 Hz (MUA) power responses in 85-ms bins from electrodes in the NOR (18 electrodes) and POR (20 electrodes) regions (the stimuli lasted for 2 s). The first 15 ms of each 100-ms data containing the electrical artifact was removed. The data were averaged over 200 trials. (**f**−**g**) Distributions of the MUA and LFP power changes (mean power within 2 s) in the NOR (119 electrodes) and POR (134 electrodes) regions from 6 cats.

Imaging with OIS mainly reflects the signals in the upper cortex due to the limitation of light penetration. To determine the planar differences in neuronal activities, we performed planar electrophysiological recordings with UEA in cortical depth of 500–600 μm in 6 cats. The OIS image at 530 nm was used to identify the POR and NOR regions to which UEA could subsequently be inserted. Figure 5a shows activation maps by TcES (10 Hz, 1.2 mA, 3 ms) and the insertion locations of the UEA. The POR (warm colors) and NOR (cold colors) regions were indicated as significantly activated pixels by TcES in 530-nm image, which can be used to determine the electrodes within the POR and NOR regions. The relative LFP and MUA power responses in the two regions were calculated and shown in Fig. 5b–e (see Methods). In the NOR region, there were total 18 electrodes and almost all of these recording sites displayed increased LFP power except for one, which showed no significant response (response amplitude <3 SD was considered as no significant response change) (Fig. 5b). However, this is not the case for MUA power. Significantly decreased MUA power was recorded from 7 out of 18 electrodes, while 8 electrodes showed increased MUA power and 3 displayed no significant change of the MUA power (Fig. 5c). In the POR region, there were total 20 electrodes and all of them displayed increased LFP power (Fig. 5d). Most electrodes (18 out of 20) exhibited increased MUA power, except one showed decreased and another one showed no significant response (Fig. 5e). The distributions of LFP and MUA power amplitudes in electrodes within the POR and NOR regions from 6 cats were shown in Fig. 5f,g. Compared to the POR region, most of the electrodes in the NOR region showed negative or positive with low-amplitude MUA and LFP power.

The numbers of electrodes for neuronal activity changes from each cat were shown in Table 1. The percentages of electrodes with increased, decreased and no significant response were calculated for each cat. For increased MUA power, the percentage of electrodes in the NOR region was significantly less (36.060 ± 5.314% vs. 71.593 ± 5.543%, Mann–Whitney test, *P* = 0.004, n = 6) than that in the POR region. For decreased MUA power, the percentage of electrodes in the NOR region was significantly larger (37.773 ± 3.296% vs. 8.418 ± 2.877%, Mann–Whitney test, *P* = 0.004, n = 6) than that in the POR region. No significant (26.167 ± 3.258% vs. 20.022 ± 3.748%, Mann–Whitney test, *P* = 0.199, n = 6) differences in percentage of electrodes with no MUA power change were found between the NOR and POR regions. For increased LFP power, the percentage of electrodes in the NOR region was less (90.042 ± 3.100% vs. 97.688 ± 1.036%, Mann–Whitney test, *P* = 0.028, n = 6) than that in the POR region. The differences in the percentage of electrodes with no LFP power change were also significant (9.875 ± 3.125% vs. 2.213 ± 0.993%, Mann–Whitney test, *P* = 0.028, n = 6) between the NOR and POR regions.

In addition, the amplitudes of enhanced MUA power recorded in the NOR regions were significantly less than those in the POR regions (7.4 ± 0.6% vs. 35.0 ± 3.4%, Mann–Whitney test, *P* = 3.3 × 10^−6^). And the amplitudes of suppressed MUA power in the NOR regions were significantly larger than those in the POR regions (−12.5 ± 0.9% vs. −9.7 ± 3.6%, Mann–Whitney test, *P* = 0.019). The amplitudes of increased LFP power recorded in the NOR regions were significantly lower than those in the POR regions (274.5 ± 39.9% vs. 456.7 ± 54.4%, Mann–Whitney test, *P* = 7.3 × 10^−9^).

### Laminar LFP and MUA recordings by linear microelectrode array

**Figure 6:**
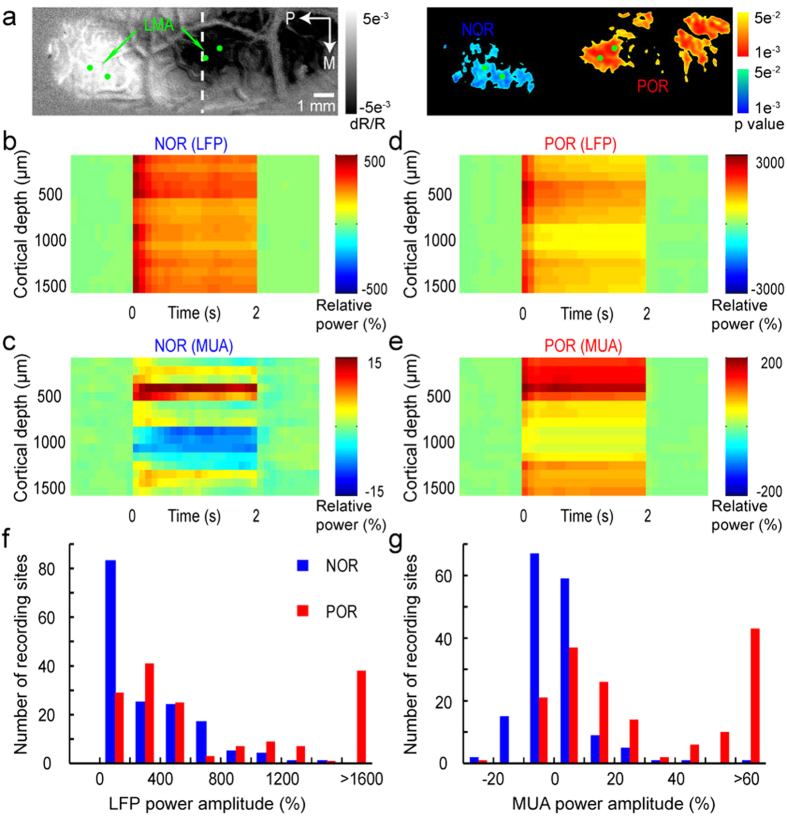
LMA recordings of MUA and LFP by TcES (10 Hz, 1.2 mA, 3 ms) in one cat. (**a**) Optical imaging map (530 nm) and corresponding *P*-value map (two-tailed t test, *P* < 0.05, n = 148 trials) of cortical activation. Green dots, locations of the LMA. (**b**−**e**) 30–130 Hz (LFP) and 300–2500 Hz (MUA) power response in 85-ms bins from LMA in the NOR and POR regions (the stimuli lasted for 2 s). The first 15 ms of each 100-ms data containing the electrical artifact was removed. The data were averaged over 200 trials. (**f**−**g**) Distributions of the MUA and LFP power changes (mean power within 2 s) in the NOR and POR regions from 5 cats (two insertion locations for each response region in one cat, 160 recording sites in total inside each response region in 5 cats).

To determine the laminar differences in neuronal activities throughout the cortical depth, two 16-channel LMA probes were inserted to the POR and NOR regions identified by OIS image at 530 nm. Neuronal activities from the POR and NOR regions were simultaneously recorded by the two LMA probes. Figure 6a shows activation maps by TcES (10 Hz, 1.2 mA, 3 ms) and the insertion locations of the LMA probes (two for each region) in the same animal as used in Fig. 5. In the POR region, the power enhancement for both LFP and MUA was evident during the stimulation period (0–2 s) (Fig. 6d,e). In the NOR region, the LFP power was increased in all the layers (Fig. 6b). However, there was a decrease of the MUA power in cortical depth of ~900–1300 μm (recording channels 9–13) (Fig. 6c). Data from all the 5 cats showed similar phenomenon of these response power changes. Figure 6f,g show the distributions of the LFP and MUA power changes by counting the number of recording sites according to the power changes (totally 160 recording sites in 5 cats). The LFP power responses recorded from most sites were significantly increased (response amplitude >3 SD were considered as significant response change) in both the NOR and POR regions. However, the LFP response amplitudes in the NOR region were significantly lower than those in the POR region (321.6 ± 25.1% vs. 1009.8 ± 80.8%, Mann–Whitney test, *P* = 4.2 × 10^−14^). In deep cortical depth of ~900–1300 μm (channels 9–13), the numbers of recording sites (totally 50 sites) with significantly increased and decreased MUA responses in the POR region were 34 and 8 while those in the NOR region were 7 and 23. In cortical depth of ~500–700 μm (channels 5–7) which was similar to the depth of UEA recordings, the numbers of recording sites (totally 30 sites) with significantly increased and decreased MUA responses in the POR region were 20 and 5 while those in the NOR region were 9 and 10. Similar to the LFP response, the amplitudes of increased MUA response in the NOR region were significantly lower than those in the POR region (13.2 ± 3.0% vs. 77.8 ± 11.2%, Mann–Whitney test, *P* = 1.6 × 10^−11^).

## Discussion

In the present study, we found an antagonistic spatial pattern of OIS response evoked by TcES. High-resolution imaging of CBF with LSCI revealed a significant CBF decrease in the NOR region and an increase in the POR region, respectively. The onset and peak times of the negative hemodynamic responses (including oximetric, volumetric and CBF signals) were always delayed than those of the positive hemodynamic responses. By using planar and laminar electrophysiological recordings, we advance our understanding of the neuronal mechanisms underlying the negative hemodynamic responses. Our results suggest that the NOR observed here is due to decreased CBF and neuronal activities in the NOR region are not all decreased.

TcES evoked an antagonistic cortical activation, which means an opposite hemodynamic change was found adjacent to traditional hemodynamic response regions. In the visual cortex, however, this antagonistic response pattern elicited by electrical stimulation of the intact retina is first reported. Exploring the origin of the antagonistic cortical activation with multimodal approaches can provide implications of the cortical response following the retinal electrical stimulation and contribute to elucidating the physiological basis of the negative BOLD response.

We measured the changes in volumetric and oximetric signals simultaneously by using the OIS imaging. For volumetric signal, a large decrease (CBV increase) in light reflectance in the POR region was always accompanied by a corresponding reflectance increase (CBV decrease) in adjacent NOR region after the stimulus onset. Similarly, the oximetric signal in the POR region exhibited an initial dip in light reflectance followed by a large overshoot, while in the NOR region it showed a transient increase followed by a large undershoot. It is commonly thought that the initial dip at oximetric wavelengths is caused by local deoxygenation prior to a delayed vascular response^37^. However, recent studies have shown that the initial dip largely reflects an early increase in CBV not a measure of deoxygenation^34^,^38^,^39^,^40^,^41^,^42^. Our results revealed that the volumetric signal had a quick response similar to the oximetric signal. We furthermore found high correlation between the volumetric and oximetric responses in both the POR and NOR regions, which indicates a close coupling between them. Therefore, the early change of CBV played an important role in the initial change in oximetric signals. In the NOR region, it is rational that the initial increase of signals at wavelengths of 610 and 630 nm was induced by a rapid CBV decrease.

In the POR region, the overshoot part following the initial dip in oximetric signal is considered as an equivalence to the positive BOLD response^38^,^43^,^44^. In the NOR region, The undershoot part following the initial transient increase is relevant to the negative BOLD response^45^ and may be caused by an overcompensation of decreased CBV and CBF for the reduction in energy consumption due to suppressed neuronal activity^19^,^20^,^21^,^45^. Decreases in CBV and CBF had been confirmed in our study. The initial transient increase in oximetric signal has been observed in studies on negative OIS^8^,^15^,^46^ and negative BOLD responses^47^. However, there is no initial increase in most of the reported negative BOLD signals. A possible reason is that BOLD fMRI may not be able to detect such weak signal because the initial dip of the POR, which is several times larger than the initial increase of the NOR, cannot either be detected in many BOLD fMRI studies^37^. OIS can be used to accurately measure the negative BOLD phenomenon^33^. Its high spatiotemporal resolution makes it possible to detect this weak signal.

Although the inverted OIS response has been reported previously, the spatiotemporal characteristics evoked by the electrical stimulation of visual system and its correlation with the stimulation are still unknown. By using BOLD fMRI, Klingner *et al.*^17^ found the amplitude of the negative hemodynamic response was monotonically changed with current intensity of electrical stimulus in human somatosensory cortex. We analyzed the NOR evoked by the TcES with different pulse widths. Compared with the POR, activation area and amplitude of the NOR first increased with the increment of pulse width then decreased. The strength of the NOR was about one-third to one-half of those of the POR, which is in agreement with previous studies^22^,^48^. The NOR had later onset and peak times than those of the POR, which are also in accordance with previous studies^28^,^47^,^49^. All these differences between the POR and NOR suggested that their mechanisms elicited by TcES might be different. Negative BOLD and OIS responses were always related to a decrease in CBF^12^,^15^,^18^,^23^,^26^. In order to explore whether the NOR observed here was due to decreased CBF, we directly measured CBF change in both the POR and NOR regions using LSCI. We observed a significant increase in CBF in the POR region, on the contrary, a significantly decreased and delayed CBF was detected in the NOR region. This provides a direct evidence that the NOR is associated with the decreased CBF.

Imaging with OIS mainly reflects the signals in the upper cortex due to the limitation of light penetration^50^,^51^. To reveal the neural mechanism underlying the OIS response, the planar LFP and MUA were recorded by UEA which was inserted into the cortex at a depth of ~500–600 μm. It is generally known that the positive OIS and BOLD responses were coupled to enhanced neural activity. This is also confirmed by our quantitative spectral power analysis which showed large increases in LFP and MUA power responding to the TcES in the POR region. Although the negative hemodynamic response has been commonly reported in the OIS and fMRI studies, its origin remains controversial^2^,^5^,^32^. There is growing evidence that the negative hemodynamic response was related with suppressed neuronal activity^7^,^19^,^22^,^52^ or enhanced neuronal inhibition without a change in spontaneous activity^20^. However, the negative hemodynamic response associated with increased neural activity has also been reported^15^,^53^,^54^. In this study, both increased (~36% of electrodes) and decreased (~38% of electrodes) MUA responses to the TcES were detected in the NOR region by the UEA recording. The number of electrodes with decreased MUA power recorded in the NOR region was significantly more than that in the POR region. Besides, the amplitudes of increased MUA power in the NOR region were also significantly lower. In rat visual cortices, Yin *et al.*^52^ also demonstrated that the negative hemodynamic response was associated with decreased spike activities in the cortical depth of approximately 500 μm. LFP responses from UEA were all increased in the POR and NOR regions, however, the amplitude of LFP response in the NOR region was significantly smaller than that in the POR region. These results are in agreement with previous studies^19^,^20^.

By using LMA or single electrode, some studies have reported either decreased or increased neuronal activity in the negative hemodynamic response region^15^,^19^,^22^. We also tried to determine the laminar differences in neuronal activities in the POR and NOR regions. Similar to the results of UEA recordings, we recorded both the decreased and increased neuronal activities in the cortical depth of 500–700 μm in the NOR region. The advantage of planar array is that it allows multichannel recordings simultaneously in the response region. According to the results of UEA recordings, the decreased and increased neuronal activities were coexisted in the cortical depth of 500–600 μm in the NOR region. Our results provide direct evidence that not all the activity of neurons in the negative hemodynamic response region is suppressed. Inhibition is ubiquitous in visual cortical neurons and stimulus-evoked hemodynamic signals are dependent on the interactions between neuronal excitation and inhibition^2^. Compared to the POR region, the greater inhibitory activity in the NOR region may change the balance between excitatory and inhibitory effects and produce a net inhibition^2^,^45^,^55^. The cortical depth of 500–600 μm belongs to the layer 4 which has the most dense vascularization^56^. A growing body of literature shows that hemodynamic response in the brain is regulated by neurons and astrocytes which can act as intermediaries to these neuronal populations^3^,^57^. Both Neuron- and astrocyte-derived vasoactive messengers exert direct effects on the microcirculation^3^. Variations in the balance between neuronal inhibition and excitation may change proportion between released vasoconstrictors and vasodilators by neurons and astrocytes, which may contribute to a net negative vascular response change^2^.

Our study is the first reporting TcES-induced neuronal inhibition but its origin is still unclear. The negative hemodynamic response in visual cortices has been investigated by using different visual stimulation, annular visual stimulus is the most common way^10^,^22^,^23^,^26^,^29^. In these studies, the positive hemodynamic response mainly occurred in visual cortex involved in the stimulus processing while the negative hemodynamic response in the surrounding regions. Due to the ring-like structure of ERG-jet electrode, through which TcES preferentially activates peripheral retina and may have an annular stimulation effect similar to the annular visual stimulus. In our study the NOR was found more posterior than the POR in the exposed visual cortex. Based on our previous study, the POR region was located mainly in cortical areas representing peripheral visual field since TcES preferentially activated peripheral retina^36^. Hence, the mechanisms of TcES-induced deactivation may be similar to those of negative response evoked by annular visual stimulus, such as reduced feedback input from higher visual areas, reduced feedforward excitation from the LGN or feedback-mediated inhibition via GABAergic interneurons^26^. Besides, Electrical stimulation of visual cortical afferents could disrupt cortico-cortical signal propagation and induce strong cortical inhibition^30^. Therefore, TcES-evoked inhibitory activity may be also related with the disruption of cortico-cortical signal propagation. The ideal experimental design should use natural stimulation, however, the mechanisms underlying the negative hemodynamic response has been studied by many other research groups using electrical stimulation^16^,^17^,^18^,^19^. Devor *et al.*^14^,^20^ demonstrated that the positive and negative hemodynamic pattern in primary somatosensory cortex evoked by electrical forepaw stimulus is consistent with those evoked by natural single-vibrissa stimulus. Our results using the TcES could provide some important indications about the underpinning of the inverted optical signal.

In conclusion, an antagonistic spatial pattern of activation in cat visual cortex evoked by TcES was reported for the first time. Compared with other functional imaging methods, optical imaging has higher spatial resolution and could provide some indications of mechanisms underlying the negative hemodynamic response. With the increment of pulse width, the POR was monotonically increased while the NOR was increased firstly and then decreased. Both the onset and peak times of negative hemodynamic responses were later than those of positive hemodynamic responses including the oximetric, volumetric and CBF signals. Decreased CBV may induce the initial increase of the oximetric signal in the NOR region. The negative hemodynamic response reported here does not imply decreased activity in all neurons. The evidence of both increased and decreased neuronal activities in the negative response region suggests that careful attention should be paid in interpreting the negative OIS or BOLD response.

## Methods

### Animal preparations

All experimental procedures were performed with approval from the Ethics Committee of Shanghai Jiao Tong University and were in accordance with the ARVO Statement on the Use of Animals in Ophthalmic and Vision Research and the National Institute of Health (NIH) Principles of Laboratory Animal Care.

Thirty-one healthy male adult cats (Fengxian, Shanghai, China) weighing 1.5–3.0 kg were prepared and maintained as described previously^36^ except that tiletamine-zolazepam (5 mg/kg, i.m.; Virbac, Carros, France) was used for initial anesthesia instead of ketamine in 18 out of 31 cats. The cats received atropine sulphate (0.15 mg/kg; Harvest Pharmaceutical Co. Ltd, Shanghai, China) and dexamethasone (25 mg/kg) to reduce mucosal secretions and cerebral edema. Anesthesia was maintained by ventilation with isoflurane (2–3% during surgery, 1–1.5% during recording; Jiupai Pharmaceutical Co. Ltd, Hebei, China) using a pulmonary pump (Model 3000, Matrx, New York, NY, USA). The femoral vein was catheterized to deliver fluids (mixture of gallamine triethiodide 10 mg/kg/h and glucose 24 mg/kg/h; Sigma-Aldrich Corp., St. Louis, MO, USA) to maintain muscle relaxation and nutrition supply. The animal eyes were covered by contact lenses for corneal protection. A water-circulating heating pad (T/Pump TP702, Gaymar Industries, New York, NY, USA) was used to maintain body temperature of the animal. Rectal temperature, end-expiratory CO_2_, blood pressure and ECG were monitored with a multi-parameter life monitor (PM-8000 Express, Mindray, Shenzhen, China) during the experiment.

After the cats mounted in a stereotaxic frame (SN-3N, Narishige, Japan), craniotomy and durotomy were performed to expose visual areas 17 and 18 (Horsley–Clarke coordinates AP −9 to +9 mm, ML 0.5 to 6 mm) at left hemisphere. A stainless steel chamber of 26-mm diameter was fixed to the skull using dental cement for optical imaging. Silicone oil (DMPS-5X, Sigma-Aldrich Corp., St. Louis, MO, USA) was filled in the chamber and covered by a round glass slip to reduce movement of the brain. The imaging system was positioned on a floating vibration isolation platform to minimize motion artifacts.

### OIS imaging and LSCI

Stimulus-induced hemodynamic signals were measured by OIS imaging in all the cats. As previously reported, a CCD camera (Dalsa 1M60, resolution 1024 × 1024 pixels, Dalsa Corporation, Waterloo, Canada) was positioned above the chamber to record the image of the visual cortex that was illuminated by a fiber optic light guide at three different wavelengths of 530 ± 10, 610 ± 10 and 630 ± 10 nm sequentially^36^. Optical intrinsic signal at ‘volumetric’ (530 nm) wavelength reflects changes in total hemoglobin (HbT) and is thought to be equivalent to the CBV due to the same absorption coefficient of oxyhemoglobin (HbO) and deoxyhemoglobin (HbR) at this wavelength^38^. Signals at oximetric (610 and 630 nm) wavelengths carry information about changes in blood oxygenation because the HbO absorbs much lower 610- and 630-nm lights than the HbR does^38^,^58^. The camera recorded 24 image frames per second, which provided an effective frame rate of 8 Hz for each of the three wavelengths. The focal plane was fixed at 500 μm under the surface of the visual cortex. Each recording trial lasted for 18 s containing 1 s before the stimulus onset for electrical stimulation.

The dynamic evolution of CBF was measured with LSCI in 5 cats. The experimental setup for the LSCI was similar to the OIS imaging except that a laser diode (L780P010, Thorlabs, Newton, NJ, USA) powered by a driver module (LDC220C, Thorlabs, Newton, NJ, USA) was used as light source and the frame rate of CCD camera operating in 2 × 2 binning mode was set at 56 Hz. Each recording trial lasted for 18 s containing 2 s before the stimulus onset for electrical stimulation.

### Electrophysiological recordings

We used Utah microelectrode array (Blackrock Microsystems, Salt Lake City, UT, USA) that contained 100 electrodes arranged in a 10 × 10 grid for planar electrophysiological recordings in 6 cats. The electrodes were 1.5 mm in length and spaced 400 μm by 400 μm. The electrode impedances were measured with a 1 kHz sinusoidal constant current signal and ranged from 300 to 800 kΩ. The array was inserted to a depth of approximately 500–600 μm in the cortex using a pneumatic inserter (Blackrock Microsystems, Salt Lake City, UT, USA). The inserted sites were designated according to previous optical imaging under 530-nm illumination.

Two linear microelectrode arrays (Micro Probe Inc., Gaithersburg, MD, USA) were used to record depth-dependent neuronal activity in 5 cats. The electrode array was a single probe (6 cm in length and 265 μm in diameter) with a tapering tungsten tip (1 mm in length) and 16 electrode contacts (25 μm in diameter, 100 μm in spacing and 300 to 500 kΩ in impedance) made of platinum/iridium. Two probes were held in place by two stereotactic micromanipulators (SM-11, Narishige, Japan) and inserted slowly perpendicular to a depth of 1600 μm positioned at center of the regions displaying the greatest increment and decrement in 530-nm OIS image elicited by TcES.

Reference wires were inserted in subdural space and ground wires were connected to a stainless steel needle penetrated into cats’ lateral scalp. To reduce cortical pulsation during the recording, the exposed cortex was covered with 2% agar. Multi-channel signals were recorded by neurophysiology workstations (RX7 and RX5, Tucker-Davis Technologies Co., Alachua, FL, USA) at a 25 kHz sampling rate and filtered at 0.3 Hz–10 kHz for the following offline processing procedure. Each recording trial consisted of stimuli of 2 s and an inter-stimulus interval (ISI) of 8 s.

### Electrical stimulation

An ERG-jet electrode (CareFusion, Middleton, WI, USA) was placed on the cornea surface of cats’ right eye instead of contact lens during electrical stimulation and a stainless steel needle inserted into dorsal neck muscle was used as return electrode. We used hydroxyethylcellulose gel (1.3%; Aqualon Co., Wilmington, DE, USA) to protect the cornea and keep good conductivity. Biphasic charge-balanced rectangular cathode-first current pulses generated by an isolated and programmable stimulating system (MS16, Tucker-Davis Technologies Co., Alachua, FL, USA) were used for the electrical stimulation. The stimulus duration in each trial was 2 s. We investigated spatiotemporal patterns (Figs 1 and 2) of the cortical activation by TcES (frequency, 20 Hz; current intensity, 1.2 mA; pulse width, 10 ms) in 16 cats. The same electrical stimulation parameters were used in 5 cats for LSCI (Fig. 4). Different electrical stimulation parameters (20 Hz, 1.2 mA, 2–22 ms) were used for unveiling the correlation between the antagonistic response and the electrical stimulation in 6 cats (Fig. 3). In order to facilitate electrophysiological data analysis, fixed stimulation parameter (10 Hz, 1.2 mA, 3 ms) was used in 10 cats for electrophysiological recording and corresponding OIS imaging (Figs 5 and 6).

### Data analysis

All data analysis was performed using user-defined MATLAB program. For OIS data analysis, video frames were subtracted and divided by a blank image (binned from −1 to −0.25-s frames), and then averaged trial-by-trial to generate images of light reflectance change (dR/R). The dR/R maps were averaged into 250-ms maps to display the time course of cortical responses. Single-condition map was created by averaging over frames during 1.75–2.25 s after stimulus onset for a given stimulus condition. Two 1.4 × 1.4 mm regions of interest (ROI), with the greatest averaged reflectance decrement and increment, were selected in single-condition maps at 530 nm and used for subsequent analysis for all images at three wavelengths. Two-tailed *t* test^59^ was conducted in dR/R maps to evaluate cortical response to TcES after smoothing (170 μm in diameter) by circular averaging filters. Pixels with significantly (*P* < 0.05) decreased and increased reflectance ratio in *P*-value maps were encoded in warm and cold colors respectively. We quantitatively determined optical response latency as the first time point where the reflectance change showed a statistically significant inflection in slope^38^. The response onset and peak times were estimated based on the camera recording time of each frame.

For the LSCI analysis, speckle contrast images were obtained by using temporal laser speckle contrast analysis algorithm^60^. Each speckle contrast image was calculated with 15 frames of raw speckle images, leading to a temporal resolution of ~0.125 s. Then images of CBF velocity were computed based on the principle that the CBF velocity varies inversely as square of speckle contrast value^60^. All the CBF velocity images were filtered with a Gaussian matrix (half width = 5 pixels) to remove spatial noises. After that the relative changes in CBF were obtained by subtracting and dividing the baseline from each CBF image.

For electrophysiological data, in order to provide more useful information on electrophysiological responses, the raw neural data were subjected to spectral power analysis^19^,^22^. For 10-Hz TcES, Fourier transform was performed on data from non-overlapping 85-ms (first 15-ms data containing electrical stimulation artifact was removed) portions of raw broadband electrophysiological signal^19^. Relative power of local field potential (LFP) and multiunit activity (MUA) bands in each bin was calculated by averaging the fractional change (relative to a baseline of 4 s) in power over the ranges of 30–130 Hz and 300–2500 Hz, and then averaged across trials.

### Statistical analyses

Data are expressed as mean ± standard error of the mean (SEM). Wilcoxon test was used for matched-pair data, Mann–Whitney test for comparing independent data between two groups. *P* < 0.05 was considered statistically significant for all the tests.

## Additional Information

**How to cite this article**: Ma, Z. *et al.* Inverted optical intrinsic response accompanied by decreased cerebral blood flow are related to both neuronal inhibition and excitation. *Sci. Rep.* **6**, 21627; doi: 10.1038/srep21627 (2016).

## Figures and Tables

**Table 1:** Numbers of electrodes for neuronal activity changes in the NOR and POR regions recorded by UEA from six cats.

	NOR region						∑	POR region						∑
	MUA			LFP				MUA			LFP			
	↓	↑	○	↓	↑	○		↓	↑	○	↓	↑	○	
Animal														
1	11	6	8	0	21	4	25	4	12	5	0	20	1	21
2	7	9	8	0	19	5	24	0	19	4	0	22	1	23
3	7	8	3	0	17	1	18	1	18	1	0	20	0	20
4	5	7	4	0	15	1	16	3	12	6	0	20	1	21
5	9	3	6	0	16	2	18	1	16	7	0	24	0	24
6	6	9	3	0	18	0	18	2	19	4	0	25	0	25

↓, decreased power response; ↑, increased power response; ○, no significant power change; ∑, total numbers of electrodes in the POR or NOR regions. Response amplitude >3 SD was considered as significant change.

## References

[b1] Logothetis N. K., Pauls J., Augath M., Trinath T. & Oeltermann A. Neurophysiological investigation of the basis of the fMRI signal. Nature 412, 150–157 (2001). 11449264 10.1038/35084005

[b2] Lauritzen M., Mathiesen C., Schaefer K. & Thomsen K. J. Neuronal inhibition and excitation, and the dichotomic control of brain hemodynamic and oxygen responses. Neuroimage 62, 1040–1050 (2012). 22261372 10.1016/j.neuroimage.2012.01.040

[b3] Cauli B. & Hamel E. Revisiting the role of neurons in neurovascular coupling. Front. neuroenerg. 2, 9, (2010). 10.3389/fnene.2010.00009 PMC2899521 20616884

[b4] Zapeda A., Arias C. & Sengpiel F. Optical imaging of intrinsic signals: recent developments in the methodology and its applications. J. Neurosci. Methods 136, 1–21 (2004). 15126041 10.1016/j.jneumeth.2004.02.025

[b5] Hayes D. J. & Huxtable A. G. Interpreting deactivations in neuroimaging. Front. Psychol. 3, 27 (2012). 22347207 10.3389/fpsyg.2012.00027 PMC3273719

[b6] Moraschi M., DiNuzzo M. & Giove F. On the origin of sustained negative BOLD response. J. neurophysiol. 108, 2339–2342 (2012). 22723671 10.1152/jn.01199.2011

[b7] Boorman L. *et al.* Long-Latency Reductions in Gamma Power Predict Hemodynamic Changes That Underlie the Negative BOLD Signal. J. Neurosci. 35, 4641–4656 (2015). 25788681 10.1523/JNEUROSCI.2339-14.2015 PMC4363390

[b8] Maggioni E. *et al.* Investigation of negative BOLD responses in human brain through NIRS technique. A visual stimulation study. Neuroimage 108, 410–422 (2015). 25576645 10.1016/j.neuroimage.2014.12.074

[b9] Puckett A. M., Mathis J. R. & DeYoe E. A. An investigation of positive and inverted hemodynamic response functions across multiple visual areas. Hum. Brain Mapp. 35, 5550–5564 (2014). 25044672 10.1002/hbm.22569 PMC4254893

[b10] Huber L. *et al.* Investigation of the neurovascular coupling in positive and negative BOLD responses in human brain at 7 T. Neuroimage 97, 349–362 (2014). 24742920 10.1016/j.neuroimage.2014.04.022

[b11] Martin C., Zheng Y., Sibson N. R., Mayhew J. E. & Berwick J. Complex spatiotemporal haemodynamic response following sensory stimulation in the awake rat. Neuroimage 66, 1–8 (2013). 23063446 10.1016/j.neuroimage.2012.10.006 PMC3556776

[b12] Stefanovic B., Warnking J. M. & Pike G. B. Hemodynamic and metabolic responses to neuronal inhibition. Neuroimage 22, 771–778 (2004). 15193606 10.1016/j.neuroimage.2004.01.036

[b13] Hamzei F. *et al.* Reduction of excitability (“inhibition”) in the ipsilateral primary motor cortex is mirrored by fMRI signal decreases. NeuroImage 17, 490–496 (2002). 12482101 10.1006/nimg.2002.1077

[b14] Devor A. *et al.* Coupling of the cortical hemodynamic response to cortical and thalamic neuronal activity. Proc. Natl. Acad. Sci. USA 102, 3822–3827 (2005). 15734797 10.1073/pnas.0407789102 PMC550644

[b15] Devor A. *et al.* Stimulus-induced changes in blood flow and 2-deoxyglucose uptake dissociate in ipsilateral somatosensory cortex. J. Neurosci. 28, 14347–14357 (2008). 19118167 10.1523/JNEUROSCI.4307-08.2008 PMC2655308

[b16] Kastrup A. *et al.* Behavioral correlates of negative BOLD signal changes in the primary somatosensory cortex. Neuroimage 41, 1364–1371 (2008). 18495495 10.1016/j.neuroimage.2008.03.049

[b17] Klingner C. M., Hasler C., Brodoehl S. & Witte O. W. Dependence of the negative BOLD response on somatosensory stimulus intensity. Neuroimage 53, 189–195 (2010). 20538064 10.1016/j.neuroimage.2010.05.087

[b18] Schafer K. *et al.* Negative BOLD signal changes in ipsilateral primary somatosensory cortex are associated with perfusion decreases and behavioral evidence for functional inhibition. Neuroimage 59, 3119–3127 (2012). 22155327 10.1016/j.neuroimage.2011.11.085

[b19] Boorman L. *et al.* Negative blood oxygen level dependence in the rat: a model for investigating the role of suppression in neurovascular coupling. J. Neurosci. 30, 4285–4294 (2010). 20335464 10.1523/JNEUROSCI.6063-09.2010 PMC6634501

[b20] Devor A. *et al.* Suppressed neuronal activity and concurrent arteriolar vasoconstriction may explain negative blood oxygenation level-dependent signal. J. Neurosci. 27, 4452–4459 (2007). 17442830 10.1523/JNEUROSCI.0134-07.2007 PMC2680207

[b21] Harel N., Lee S. P., Nagaoka T., Kim D. S. & Kim S. G. Origin of negative blood oxygenation level-dependent fMRI signals. J. Cereb. Blood Flow Metab. 22, 908–917 (2002). 10.1097/00004647-200208000-00002 12172376

[b22] Shmuel A., Augath M., Oeltermann A. & Logothetis N. K. Negative functional MRI response correlates with decreases in neuronal activity in monkey visual area V1. Nat. Neurosci. 9, 569–577 (2006). 16547508 10.1038/nn1675

[b23] Shmuel A. *et al.* Sustained negative BOLD, blood flow and oxygen consumption response and its coupling to the positive response in the human brain. Neuron 36, 1195–1210 (2002). 12495632 10.1016/s0896-6273(02)01061-9

[b24] Wade A. R. & Rowland J. Early Suppressive Mechanisms and the Negative Blood Oxygenation Level-Dependent Response in Human Visual Cortex. J. Neurosci. 30, 5008–5019 (2010). 20371821 10.1523/JNEUROSCI.6260-09.2010 PMC3523120

[b25] Smith A. T., Williams A. L. & Singh K. D. Negative BOLD in the visual cortex: evidence against blood stealing. Hum. Brain Mapp. 21, 213–220 (2004). 15038003 10.1002/hbm.20017 PMC6871689

[b26] Pasley B. N., Inglis B. A. & Freeman R. D. Analysis of oxygen metabolism implies a neural origin for the negative BOLD response in human visual cortex. Neuroimage 36, 269–276 (2007). 17113313 10.1016/j.neuroimage.2006.09.015 PMC2001204

[b27] Zappe A. C., Uludag K. & Logothetis N. K. Direct measurement of oxygen extraction with fMRI using 6% CO2 inhalation. Magn. Reson. Imaging 26, 961–967 (2008). 18450401 10.1016/j.mri.2008.02.005

[b28] Mullinger K. J., Mayhew S. D., Bagshaw A. P., Bowtell R. & Francis S. T. Evidence that the negative BOLD response is neuronal in origin: A simultaneous EEG-BOLD-CBF study in humans. Neuroimage 94, 263–274 (2014). 24632092 10.1016/j.neuroimage.2014.02.029

[b29] Goense J., Merkle H. & Logothetis N. K. High-Resolution fMRI Reveals Laminar Differences in Neurovascular Coupling between Positive and Negative BOLD Responses. Neuron 76, 629–639 (2012). 23141073 10.1016/j.neuron.2012.09.019 PMC5234326

[b30] Logothetis N. K. *et al.* The effects of electrical microstimulation on cortical signal propagation. Nat. Neurosci. 13, 1283–1291 (2010). 20818384 10.1038/nn.2631

[b31] Sutan F., Augath M., Murayama Y., Tolias A. S. & Logothetis N. esfMRI of the upper STS: further evidence for the lack of electrically induced polysynaptic propagation of activity in the neocortex. Magn. Reson. Imaging 29, 1374–1381 (2011). 21757310 10.1016/j.mri.2011.04.005

[b32] Kim S. G. & Ogawa S. Biophysical and physiological origins of blood oxygenation level-dependent fMRI signals. J. Cereb. Blood Flow Metab. 32, 1188–1206 (2012). 10.1038/jcbfm.2012.23 PMC3390806 22395207

[b33] Kennerley A. J., Mayhew J. E., Boorman L., Zheng Y. & Berwick J. Is optical imaging spectroscopy a viable measurement technique for the investigation of the negative BOLD phenomenon? A concurrent optical imaging spectroscopy and fMRI study at high field (7 T). Neuroimage 61, 10–20 (2012). 22440642 10.1016/j.neuroimage.2012.03.015 PMC3368428

[b34] Hillman E. M. Coupling mechanism and significance of the BOLD signal: a status report. Annu. Rev. Neurosci. 37, 161–181 (2014). 25032494 10.1146/annurev-neuro-071013-014111 PMC4147398

[b35] Guillory K. S. & Normann R. A. A 100-channel system for real time detection and storage of extracellular spike waveforms. J. Neurosci. Methods 91, 21–29 (1999). 10522821 10.1016/s0165-0270(99)00076-x

[b36] Ma Z. *et al.* Optical imaging of visual cortical responses evoked by transcorneal electrical stimulation with different parameters. Invest. Ophthalmol. Vis. Sci. 55, 5320–5331 (2014). 25082881 10.1167/iovs.14-14600

[b37] Hu X. P. & Yacoub E. The story of the initial dip in fMRI. Neuroimage 62, 1103–1108 (2012). 22426348 10.1016/j.neuroimage.2012.03.005 PMC3389272

[b38] Sirotin Y. B., Hillman E. M., Bordier C. & Das A. Spatiotemporal precision and hemodynamic mechanism of optical point spreads in alert primates. Proc. Natl. Acad. Sci. USA 106, 18390–18395 (2009). 19828443 10.1073/pnas.0905509106 PMC2775289

[b39] Chen B. R., Bouchard M. B., McCaslin A. F., Burgess S. A. & Hillman E. M. High-speed vascular dynamics of the hemodynamic response. Neuroimage 54, 1021–1030 (2011). 20858545 10.1016/j.neuroimage.2010.09.036 PMC3018836

[b40] Martin C., Martindale J., Berwick J. & Mayhew J. Investigating neural-hemodynamic coupling and the hemodynamic response function in the awake rat. Neuroimage 32, 33–48 (2006). 16725349 10.1016/j.neuroimage.2006.02.021

[b41] Das A. & Sirotin Y. B. Reply to Uludag: fMRI “initial dip” reflects increase in oxygenated hemoglobin. Proc. Natl. Acad. Sci. USA 107, E24–E24 (2010).

[b42] Sheth S. A. *et al.* Linear and nonlinear relationships between neuronal activity, oxygen metabolism, and hemodynamic responses. Neuron 42, 347–355 (2004). 15091348 10.1016/s0896-6273(04)00221-1

[b43] Ogawa S., Lee T. M., Kay A. R. & Tank D. W. Brain magnetic resonance imaging with contrast dependent on blood oxygenation. Proc. Natl. Acad. Sci. USA 87, 9868–9872 (1990). 2124706 10.1073/pnas.87.24.9868 PMC55275

[b44] Suh M., Bahar S., Mehta A. D. & Schwartz T. H. Blood volume and hemoglobin oxygenation response following electrical stimulation of human cortex. Neuroimage 31, 66–75 (2006). 16480899 10.1016/j.neuroimage.2005.11.030

[b45] Klingner C. M., Brodoehl S. & Witte O. W. The importance of the negative blood-oxygenation-level-dependent (BOLD) response in the somatosensory cortex. Rev. Neurosci. 26, 647–653 (2015). 26057216 10.1515/revneuro-2015-0002

[b46] Watanabe H., Homae F. & Taga G. Activation and deactivation in response to visual stimulation in the occipital cortex of 6-month-old human infants. Dev. Psychobiol. 54, 1–15 (2012). 21594872 10.1002/dev.20569

[b47] Klingner C. M. *et al.* Functional Deactivations: Multiple Ipsilateral Brain Areas Engaged in the Processing of Somatosensory Information. Hum. Brain Mapp. 32, 127–140 (2011). 21157879 10.1002/hbm.21006 PMC6870510

[b48] Hlushchuk Y. & Hari R. Transient suppression of ipsilateral primary somatosensory cortex during tactile finger stimulation. J. Neurosci. 26, 5819–5824 (2006). 16723540 10.1523/JNEUROSCI.5536-05.2006 PMC6675271

[b49] Bagshaw A. P. *et al.* EEG-fMRI of focal epileptic spikes: Analysis with multiple haemodynamic functions and comparison with gadolinium-enhanced MR angiograms. Hum. Brain Mapp. 22, 179–192 (2004). 15195285 10.1002/hbm.20024 PMC6871989

[b50] Tian P. F., Devor A., Sakadzic S., Dale A. M. & Boas D. A. Monte Carlo simulation of the spatial resolution and depth sensitivity of two-dimensional optical imaging of the brain. J. Biomed. Opt. 16, 016006–1-016006–13 (2011). 10.1117/1.3533263 PMC3041814 21280912

[b51] Hillman E. M. C. Optical brain imaging *in vivo*: techniques and applications from animal to man. J. Biomed. Opt. 12, 051402–1-051402–28 (2007). 10.1117/1.2789693 PMC2435254 17994863

[b52] Yin H. B., Liu Y. D., Li M. & Hu D. W. Hemodynamic observation and spike recording explain the neuronal deactivation origin of negative response in rat. Brain Res. Bull. 84, 157–162 (2011). 21147201 10.1016/j.brainresbull.2010.12.004

[b53] Choi J. K., Chen Y. I., Hamel E. & Jenkins B. G. Brain hemodynamic changes mediated by dopamine receptors: Role of the cerebral microvasculature in dopamine-mediated neurovascular coupling. Neuroimage 30, 700–712 (2006). 16459104 10.1016/j.neuroimage.2005.10.029

[b54] Shih Y. Y. *et al.* A new scenario for negative functional magnetic resonance imaging signals: endogenous neurotransmission. J. Neurosci. 29, 3036–3044 (2009). 19279240 10.1523/JNEUROSCI.3447-08.2009 PMC6666445

[b55] Klingner C. M., Hasler C., Brodoehl S. & Witte O. W. Excitatory and inhibitory mechanisms underlying somatosensory habituation. Hum. Brain Mapp. 35, 152–160 (2014). 22847930 10.1002/hbm.22163 PMC6869727

[b56] Logothetis N. K. & Wandell B. A. Interpreting the BOLD signal. Annu. Rev. Physiol. 66, 735–769 (2004). 14977420 10.1146/annurev.physiol.66.082602.092845

[b57] Schummers J., Yu H. B. & Sur M. Tuned responses of astrocytes and their influence on hemodynamic signals in the visual cortex. Science 320, 1638–1643 (2008). 18566287 10.1126/science.1156120

[b58] Sirotin Y. B., Cardoso M., Lima B. & Das A. Spatial homogeneity and task-synchrony of the trial-related hemodynamic signal. Neuroimage 59, 2783–2797 (2012). 22036678 10.1016/j.neuroimage.2011.10.019 PMC3254827

[b59] Tanigawa H., Lu H. D. & Roe A. W. Functional organization for color and orientation in macaque V4. Nat. Neurosci. 13, 1542–1548 (2010). 21076422 10.1038/nn.2676 PMC3005205

[b60] Boas D. A. & Dunn A. K. Laser speckle contrast imaging in biomedical optics. J. Biomed. Opt. 15, 011109–1-011109–12 (2010). 10.1117/1.3285504 PMC2816990 20210435

